# The Neural Progenitor Cell-Associated Transcription Factor FoxG1 Regulates Cardiac Epicardial Cell Proliferation

**DOI:** 10.1155/2024/8601360

**Published:** 2024-01-11

**Authors:** Lucy Pilcher, Lara Solomon, Julie A. Dragon, Dhananjay Gupta, Jeffrey L. Spees

**Affiliations:** ^1^Department of Medicine, Cardiovascular Research Institute, University of Vermont, Colchester, VT 05446, USA; ^2^Cellular and Molecular Biomedical Sciences Program, University of Vermont, Burlington, VT 05401, USA; ^3^Vermont Integrative Genomics Resource, University of Vermont Larner College of Medicine, Burlington, VT 05405, USA; ^4^Division of Endocrinology, Diabetes, and Metabolism, Department of Medicine, Larner College of Medicine, University of Vermont, Burlington, VT 05446, USA

## Abstract

The epicardium is a layer of mesothelial cells that covers the surface of the heart. During development, epicardial cells undergo epithelial-to-mesenchymal transition (EMT) to form multipotent precursors that migrate into the heart and contribute to the coronary vasculature by differentiating into adventitial fibroblasts, smooth muscle cells, and endothelial cells. Epicardial cells also provide paracrine signals to cardiac myocytes that are required for appropriate heart growth. In adult hearts, a similar process of epicardial cell EMT, migration, and differentiation occurs after myocardial infarction (MI, heart attack). Pathological cardiac hypertrophy is associated with fibrosis, negative remodeling, and reduced cardiac function. In contrast, aerobic exercises such as swimming and running promote physiological (i.e., beneficial) hypertrophy, which is associated with angiogenesis and improved cardiac function. As epicardial cell function(s) during physiological hypertrophy are poorly understood, we analyzed and compared the native epicardial cells isolated directly from the hearts of running-exercised mice and age-matched, nonrunning littermates. To obtain epicardial cells, we enzymatically digested the surfaces of whole hearts and performed magnetic-activated cell sorting (MACS) with antibodies against CD104 (integrin *β*4). By cDNA microarray assays, we identified genes with increased transcription in epicardial cells after running exercise; these included FoxG1, a transcription factor that controls neural progenitor cell proliferation during brain development and Snord116, a small noncoding RNA that coordinates expression of genes with epigenetic, circadian, and metabolic functions. In cultured epicardial cells, shRNA-mediated FoxG1 knockdown significantly decreased cell proliferation, as well as Snord116 expression. Our results demonstrate that FoxG1 regulates epicardial proliferation, and suggest it may affect cardiac remodeling.

## 1. Introduction

During development, the cells that comprise the pro-epicardial organ form the “epicardium,” or outermost layer of the heart that covers the myocardium [1, 2]. Groups of epicardial cells undergo epithelial-to-mesenchymal transition (EMT), migrating into the heart to produce epicardial derivatives: vascular smooth muscle cells, perivascular fibroblasts, and endothelial cells that contribute to the formation of the coronary vascular plexus [2–4]. During this process, simultaneous changes in EMT-associated transcription factors, cytoskeletal proteins, cell surface receptors, and integrins allow epithelial-like epicardial cells to adopt a mesenchymal, migratory phenotype [5–7].

By contrast, adult epicardial cells are typically quiescent, but retain their competence for EMT and migration. As such, they are considered as an important potential source of cells for cardiac regeneration after injury [8]. For example, during myocardial infarction (MI), the fetal epicardial program is “re-activated,” inducing epicardial cells to lose apical–basal polarity and undergo EMT to form multipotent precursor cells (a.k.a. epicardial-derived cells, EPDCs) [9, 10]. After MI, EPDCs migrate through the subepicardium toward areas with infarction [11] and participate in repair by differentiating into cardiac fibroblasts, myofibroblasts, and vascular smooth muscle cells [12].

Based on the role(s) that epicardial cells play in blood vessel formation during cardiac development and vascular repair after injury [13, 14], we hypothesized that epithelial-like epicardial cells (CD104^+^/Keratin 18^+^) contribute to remodeling associated with physiological (i.e., beneficial) hypertrophy following running exercise. Running exercise increases cardiac production of vascular endothelial growth factor (VEGF) that promotes neovascularization to enhance vascular perfusion and support myocardial hypertrophy [15, 16]. To isolate the epithelial-like epicardial cells directly from the heart surface, we applied short-term enzymatic digestion and magnetic-activated cell sorting (MACS) with antibodies to integrin *β*4 (CD104), an epitope expressed by native epicardial cells [17]. CD104^+^ epicardial cells obtained from the hearts of running-exercised mice and nonrunning (control) mice were used to compare the gene expression profiles. We found that running exercise-induced gene expression for multiple factors not previously reported in the epicardial cells, such as FoxG1 and Snord116. Through loss-of-function studies with shRNA-mediated knockdown and transgenic mice, we identified FoxG1 and Snord116 as potential targets to control the remodeling after cardiac injury or disease.

## 2. Materials and Methods

### 2.1. Running Exercise Model

C57BL/6J mice (males, 10–12 weeks of age; Jackson Laboratory, Bar Harbor, ME) were allowed to run ad libitum for 1–8 weeks before euthanization and heart harvest. Experimental mice were housed individually and provided food and water ad libitum. Each mouse cage was equipped with a running wheel and an attached odometer (CatEye America, Boulder, CO), generously provided by Dr. William Falls, Department of Psychology, University of Vermont. Running distances were recorded every 1–2 days. Age-matched control mice were housed and fed similarly, except without running wheels.

### 2.2. Epicardial Cell Isolation and Culture

In brief, hearts were perfused with PBS and excised. Epicardial cells were digested from the surface of the heart by incubation in digest buffer (HBSS supplemented with 5 mg/mL Collagenase/Dispase (Roche Diagnostics) and 10 *μ*M Cyclosporine A (Cayman Chemical Company), with gentle agitation. CD104^+^ epicardial cells were isolated by MACS using an anti-mouse CD104 antibody (AbDSerotec), anti-rat micro-beads (Miltenyi Biotech), and LS Columns (Miltenyi Biotech). Epicardial cells were cultured in DMEM/F12 with 10% fetal bovine serum or in LaSR medium (Advanced DMEM, 2.5 mM GlutaMAX and 100 *μ*g/mL ascorbic acid) with 0.5 *μ*M A83-01 on plates coated with 0.05% gelatin and 5 *μ*g/mL laminin. Detailed methods for enzymatic digestion and CD104 MACS are provided in the supplementary materials.

### 2.3. RNA Isolation and Microarray Assays

Total RNA was isolated directly after MACS with the Quick-RNA MicroPrep kit as per the manufacturer's instructions (Zymo Research Corp., Irvine, CA). Samples were pooled to help normalize: (1) variation in the number of epicardial cells isolated from individual mice (e.g., lower relative cell number isolated from controls) and (2) variation in the distance run among runners. RNA samples were submitted to the UVM Advanced Genome Technologies Core (UVM AGTC). Oligonucleotide microarray analysis of RNA expression levels was performed using Affymetrix GeneChips, Mouse Gene 2.0 ST (Affymetrix Inc., Santa Clara, CA) according to the manufacturer's protocols. In brief, an RNA input of 50 ng was used to generate cDNA through first strand and second strand synthesis reactions (Ovation® Pico WTA System V2, NuGEN). The cDNA samples were then purified using an Agencourt® RNAClean® XP magnetic bead protocol. Following purification, samples were amplified using SPIA reagents (Ovation® Pico WTA System V2, NuGEN). A final cDNA purification was performed (Agencourt® RNAClean® XP). Sample concentrations were determined with 33 *μ*g/mL/A260 constant on a Nanodrop 1000 Spectrophotometer. Approximately, 4 *μ*g of cDNA was fragmented and labeled (Encore® Biotin Module, NuGEN). Efficiency of the biotin labeling reaction was verified using NeutrAvidin (10 mg/mL) and a gel-shift assay. Samples were injected into arrays and placed into an Affymetrix Genechip® Hybridization Oven 640 at 45°C and 60 RPM for 16–18 hr. Arrays were stained using the Affymetrix Genechip® Fluidics Station 450 and scanned with the 7G Affymetrix Genechip® Scanner 3000. MicroArray data are available on ArrayExpress accession E-MTAB-10993.

### 2.4. Bioinformatics

For bioinformatic analysis, gene expression data were averaged for twosamples (*n* = 5 mice/sample), statistically filtered (cutoff of 2.0 for relative log expression (RLE)), and analyzed for quality control, based on the variation of the median and quartiles probe set intensities. Probe set statistics and identification of differential expression were performed by the Molecular Bioinformatics Shared Resource of the University of Vermont College of Medicine using Partek Genomics Suite® Version 6.6 (Partek Inc., St. Louis, MO). Probe-level intensities were calculated using the Robust Multichip Average (RMA) algorithm, including background-correction, normalization (quantile), and summarization (median polish), for each probe set and sample. Sample quality was assessed based on 3′ : 5′ ratio, RLE, and normalized unscaled standard error (NUSE). Principal component analysis (PCA) was also used to identify outlier samples that would potentially introduce latent variation into the analysis of differential expression across sample groups.

Multivariate PCA was performed on the normalized data set using the covariance matrix. Univariate linear modeling of sample groups was performed by ANOVA. The magnitude of the response (fold change calculated using the least square mean) and the *p*-value associated with each probe set and binary comparison were calculated, as well as a “step-up” adjusted *p*-value for the purpose of controlling false discovery rate [18].

Data were analyzed by the gene set enrichment (Partek), which uses a right-tailed Fisher's exact test with a null hypothesis that data are changing together strictly by chance. The alternative is that the data change in concert because they are part of a biological gene set of pathway. To perform functional clustering, the filtered gene expression data were entered into the Database for annotation, visualization, and integrated discovery (DAVID) [19] and also grouped according to gene ontology (GO) terms.

### 2.5. Immunohistochemistry and Immunocytochemistry

Hearts were perfused through the left ventricle with 5–10 mL of PBS using a 27-gauge needle, excised, rinsed in PBS, and fixed in 4% paraformaldehyde (PFA) overnight at 4°C. The hearts were then transferred to a 30% sucrose solution in PBS and incubated at 4°C until hearts had been impregnated. The hearts were embedded in OCT, rapidly frozen in 100% ethanol with dry ice, and stored at −80°C. Sections were cut at 10 *μ*m on a Cryostat rotary microtome at −27°C. Cells grown in LabTek chamber slides were fixed by a 5–10 min incubation in 4% PFA. Sections and cells were permeabilized by incubation in blocking buffer (5% goat serum and 0.1% TritonX-100 in PBS) for 1 hr at room temperature. The primary antibody was diluted into blocking buffer and incubated overnight at 4°C (unless otherwise noted). The slides were washed three times with PBS for 5 min. Secondary antibody was diluted (1 : 1000) in blocking buffer and incubated for 1 hr at room temperature. Slides were washed three times with PBS. The staining protocol was similar for all antibodies: Keratin 18 (Sigma SAB4501665, 1 : 500), Podoplanin (Santa Cruz sc-53533, 1 : 100), Gata4 (Santa Cruz 25310, 1 : 200), WT1 (Santa Cruz 192, 1 : 100), FoxG1 (Invitrogen PA5-41493, 1 : 200), proliferating cell nuclear antigen; PCNA (Cell Signaling 2586, 1 : 40000), CoxIV (Invitrogen a21348, 1 : 200), MLC2 (Santa Cruz 34490, 1 : 200), and SMA (RD systems MAB 1420, 1 : 200). Slides were mounted with Dapi-fluoromount (Southern Biotech, 010020). Imaging was done with a Leica DM600B microscope.

### 2.6. Snord116 Paternal Knockout Mouse

The Snord116 paternal knockout (Snord116p-) mouse (B6.Cg-*Snord116*^*tm1.1Uta*^/J) was generously provided by Dr. Rudolph L. Leibel. The heterozygous animals carrying the paternal deletion of Snord116 function as a knockout because the maternal allele is imprinted. This strain displays some characteristics of Prader–Willi Syndrome (PWS) including early onset postnatal growth retardation, delayed sexual maturation, increased anxiety, motor learning deficit, and hyperphagia (but not obesity) [20].

### 2.7. FoxG1 Knockdown Experiments

Rat epicardial cells were generously provided by Dr. Bader and were originally isolated by Wada et al. [21, 22]. These cells are proliferative, but to our knowledge these cells are not transformed. Rat epicardial cells with FoxG1 knockdown were generated using lentivirally transduced shRNA (Sigma, SHCLNV—TRCN0000081746). Transduction of scrambled shRNA lentiviral particles was used as a control (Sigma, SHC016V-1EA). For selection, all cells were grown in DMEM/F12 base medium containing 10% fetal bovine serum and puromycin (1 *μ*g/mL). Medium was changed every 48–72 hr. Protein was isolated using RIPA buffer and knockdown was confirmed by western blot. Protein (30 *μ*g) was run on a 10% bis–tris gel and transferred to a PVDF membrane. Ponceau S staining was used to quantify total protein prior to incubation in anti-FoxG1 (AbClonal A16851, 1 : 1000) overnight at 4°C. After washes, the blots were incubated in anti-rabbit HRP (Sigma, 1 : 2000). Following chemiluminescent detection (Pierce), blots were imaged on a LAS-4000 Imaging system (FUJIFILM).

For RT-PCR assays, total RNA was isolated from cells with an RNeasy Micro Kit (Qiagen) and treated with TurboDnase (Invitrogen). The RNA was quantified (Nanodrop ONE) and reverse transcription was carried out using a Superscript IV synthesis kit (Thermo Fisher) with random hexamer priming. qPCR was performed on a Quant Studio3 qPCR machine (Applied Biosystems) using a validated Snord116 TaqMan assay (Mm03455667_s1). *β*-Actin (Rn00667869_m1 Actb) was used to normalize loading of cDNA template. For proliferation assays, epicardial cells were plated at 50 cells/well into 96-well plates. Cell numbers were determined on Days 1, 5, and 7 using an MTS assay (Promega) and a Biotek Synergy HT plate reader (Agilent).

### 2.8. Statistics

Statistical analysis was performed with GraphPad Prism software. Values were expressed as means ± SD unless otherwise indicated. Comparisons of data from individual control and treatment groups were made by unpaired Student's *t* test. For experiments comparing multiple treatment groups, we used one-way ANOVA with post hoc testing. Values of *p* ≤ 0.05 were considered significant.

### 2.9. Ethics

All animal work was conducted in accordance with a protocol approved by the University of Vermont Institutional Animal Care and Use Committee (IACUC). Right atrial appendages were obtained from consenting cardiac bypass patients at the University of Vermont Medical Center under an IRB-approved protocol.

### 2.10. Additional Materials and Methods

Detailed protocols are provided in the supplemental material.

## 3. Results and Discussion

### 3.1. Direct Isolation of Primary Adult Keratin-18^+^ Epicardial Cells by CD104 MACS

**Figure 1:**
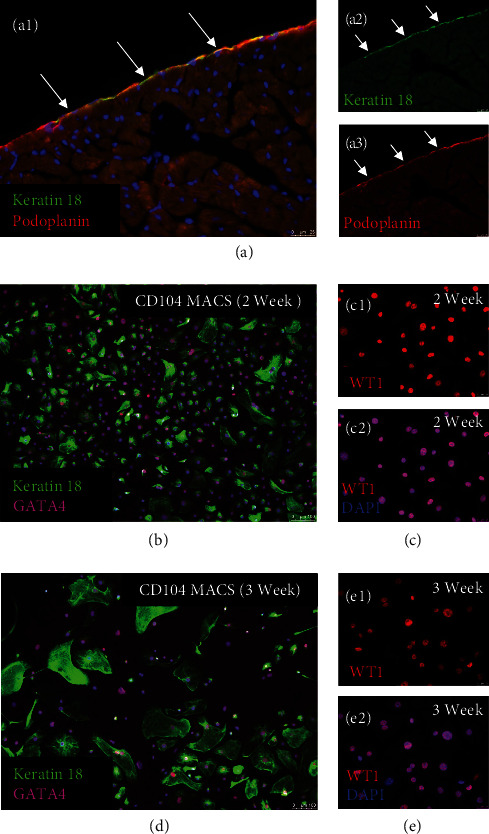
CD104 MACS enriches for Keratin-18^+^ epicardial cells. ((a): a1–a3) Keratin 18 marks podoplanin-expressing epicardial cells on the heart surface. Arrows indicate that Keratin 18 and podoplanin are localized to the epicardium. (b–e2) Epithelial-like epicardial cells were isolated by MACS using antibodies to CD104 (*β*4-integrin). Culture of CD104^+^ epicardial cells on gelatin/laminin-coated plates in LASR medium with Alk5 inhibitor prevented EMT and maintained expression of epicardial markers such as Keratin 18 and WT1 for several weeks. (b–c2) 2 weeks of culture in LASR medium. (d)–(e2) 3 weeks of culture in LASR medium.

Keratin 18 is an epithelial-associated intermediate filament protein that is expressed by podoplanin-positive epicardial cells on the heart surface, but not by other cardiac cell types (Figure 1(a)). To obtain native, epithelial-like epicardial cells for gene expression assays, we directly isolated epicardial cells from the surfaces of adult mouse hearts (C57BL/6J males, 10-week old). As some enzymes, such as trypsin, has potential to remove cell surface epitopes and receptor proteins [23–25], we used collagenase and dispase to digest ECM components [26–28]. To increase cell survival, our isolation buffer was supplemented with Cyclosporine A (CsA, 10 *μ*M), a mitochondrial transition pore inhibitor [29–31]. To further enrich epithelial-like epicardial cells, we applied MACS with antibodies directed against CD104 (integrin *β*4). Previously, Rao et al. [17] demonstrated that CD104 MACS of cells digested from the cover of the heart enriched the purity of Keratin18+ cells to 88%.

By culturing the CD104^+^ MACS fraction in a specialized medium called “LASR,” we were able to prevent the EMT of CD104^+^/Keratin 18^+^ cells and expand them for several weeks as epithelial-like cells (Figures 1(b) and 1(d)). LASR medium was originally developed by the Palecek Group to expand epithelial-like epicardial cells specified from human iPSCs [32, 33]. To inhibit EMT, LASR medium was supplemented with A83-01, an inhibitor of ALK5 (Type I transforming growth factor-*β* receptor), ALK4 (Type IB activin receptor), and ALK7 (Type I NODAL receptor). In addition to Keratin 18, cells isolated by CD104 MACS and grown in LASR medium continued to express transcription factors such as GATA4 and WT1 (Figure 1(b)–1(e2)). Of interest, we also tested LASR medium with P0 and P1 cultures of primary adult human epicardial cells isolated from right atrial appendages of cardiac bypass patients [34]. Notably, LASR medium was also effective at reducing the proportion of adult human epicardial cells that underwent EMT (Figure S1).

### 3.2. Microarray Analysis

**Figure 2:**
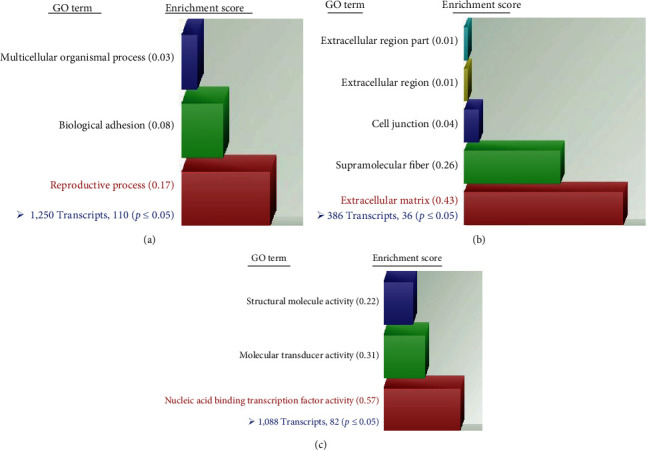
Functional clustering from DAVID analysis of microarray data. (a) Gene ontology terms identified under the GO binary filter for “Biological processes”. The highest enrichment score in this category was for “Reproductive process” (red bar). (b) Gene ontology terms identified under the GO binary filter for “Cellular processes”. The highest enrichment score in this category was for “Extracellular matrix” (red bar). (c) Gene ontology terms identified under the GO binary filter for “Molecular function”. The highest enrichment score in this category was for “Nucleic acid binding transcription factor activity” (red bar). Note: accession numbers, fold changes, and adjusted *p*-values for specific gene transcripts identified within “Reproductive process,” “Extracellular matrix,” and “Nucleic acid binding transcription factor activity” are provided in the supplemental material.

To examine the epicardial gene expression for runners and nonrunners, we pooled epicardial cells isolated by CD104 MACS from the hearts of healthy control mice and those that ran ad libitum for 1 week (*n* = 5/group). We used this early timepoint to investigate epicardial gene expression associated with cardiac remodeling. Voluntary wheel running has been shown to induce physiological cardiac hypertrophy, as measured by heart weight to body weight and cardiomyocyte cross-sectional area [35–37]. The DAVID analysis identified genes with significant changes in expression and grouped them into three major GO domains: biological process, cellular process, and molecular function. At a higher level of resolution, we examined the top GO terms identified from each of the three categories. These genes belonged to: “reproductive process,” GO:0022414; “extracellular matrix” (ECM), GO:0031012; and “nucleic acid binding transcription factor activity,” GO:0001071 (Figure 2).

#### 3.2.1. Differential Gene Expression within Gene Ontology Term “Reproductive Process”

Within the GO domain for biological processes, the GO term (GO:0022414), “reproductive,” identified 110 transcripts that significantly differed between samples (*p* ≤ 0.05; Figure 2(a), Table S1). Although, we did not see clear evidence for enhanced gene expression for endothelial cell specification of epicardial cells in our microarray data set (e.g., Scleraxis, Sema3d, Nfatc1, Etv2, and Vegfr2/Kdr), we did find changes in several important paracrine mediators of angiogenesis. We observed an increase in Endothelin 3 transcript expression (Edn3; fold change = 1.23, *p*  < 0.01) after running exercise. Under hypoxic conditions, Edn3 promotes VEGF production and endothelial cell migration [38–41]. Notably, transcription for Angiogenin-6 also increased in epicardial cells of runners (Ang6; fold change = 1.35, *p*  < 0.05). Angiogenin is a potent, 14-kDa angiogenic ribonuclease that is expressed and secreted by smooth muscle cells, endothelial cells, and stem/progenitor cells. Soluble angiogenin binds endothelial cell surface receptors, is endocytosed, and then is trafficked to the nucleus where it participates in ribosomal RNA transcription to promote endothelial cell proliferation [42–44]. Nuclear translocation of angiogenin was shown to be required for VEGF-stimulated angiogenesis [36, 45].

Several transcripts identified as differentially expressed indicated that running shifts the energetic state of epicardial cells. In epicardial cells from runners, we identified a significant decrease in gene expression (*p*  < 0.01) for the inner mitochondria membrane peptidase Immp2l (fold change = −1.41) and the outer mitochondrial membrane protein Bcl-2 (fold change = −1.27). Lu et al. [46] showed an increase in ATP production in mitochondria isolated from Immp2l-deficient mice. Bcl-2 is an antiapoptotic, prosurvival protein that forms a complex with calcineurin and inhibits mitochondrial pore transition [47, 48]. Calcineurin was shown to regulate Bcl-2 expression [49]. Although, all epicardial cells were exposed to CsA for 2 hr during our isolation procedure, it is possible that the observed difference in Bcl-2 mRNA level was due to differential responsiveness to the CsA treatment. Consistent with Wnt signaling, running-exercised mice exhibited increased transcription frizzled 9 (Fzd9; fold change = 1.50, *p* ≤ 0.01), a Wnt coreceptor that regulates proliferation and differentiation of neural progenitor cells [50].

#### 3.2.2. Differential Gene Expression within Gene Ontology Term “Extracellular Matrix”

Running induced a significant change for 36 ECM-associated genes in isolated CD104^+^ epicardial cells (*p* ≤ 0.05; Table S2). Many genes that grouped with the GO term ECM indicated that running may induce ECM degradation or modification of the epicardial basement membrane. We observed significant increases (*p* ≤ 0.05) in gene expression of the anchoring protein Ladinin1 (Lad1; fold change = 1.35) and Glypican 5 (Gpc5; fold change = 1.49), both of which regulate growth factor signal transduction [51–57]. In cancer, Lad1 downregulation during metastatic EMT contributes to overall ECM rearrangement [58, 59]. Gpc5 was shown to interact with Wnt3a and inhibit signaling through the Wnt/*β*-catenin pathway [60]. We also noted decreased gene expression (fold change = −1.73) for the matrix-assembly protein Vitrin [61]. Col7a1 was upregulated (fold change = 1.32) and contributes to matrix adhesion by anchoring collagen fibrils to adhesion molecules. Epicardial cells lacking PDGFR have reduced expression of Col7a1, which was shown to contribute to epicardial EMT failure [62–64]. By contrast, expression of Collagen 6a1 was significantly downregulated (Col6a1; fold change = −1.30). Mice deficient in Col6a1 had improved cardiac function after MI [65]. Adaptation to running exercise may require various components of the subepicardial extracellular matrix to be degraded or rearranged to allow for EPDC migration after epicardial EMT [66, 67].

#### 3.2.3. Greater Number of Epicardial Cells Isolated from Hearts of Running-Exercised Mice

**Figure 3:**
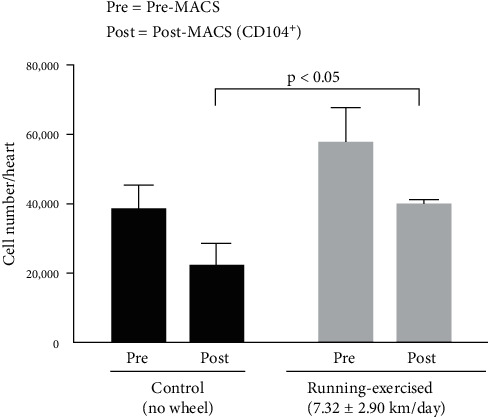
Increased number of cells isolated from hearts of running-exercised mice. Average total cell count per heart is shown for cells isolated from running-exercised mice and nonrunning littermate controls. Pre: cell number prior to CD104 MACS. Post: CD104^+^ cell number after MACS. *N* = 3 experiments, 5 hearts/experiment.

We performed cell counts after CD104 MACS and observed a significant increase in total number of cells per heart for isolates from running mice (control mice: 21,778 ± 6,500 cells/heart; running mice: 39,533 ± 1,750 cells/heart; *n* = 5 mice per group; *p*  < 0.01; Figure 3). The changes in ECM components discussed above may contribute to the increased number of cells digested and sorted from the hearts of running mice.

#### 3.2.4. Differential Gene Expression within Gene Ontology Term “Nucleic Acid Transcription Factor Binding”

We found that 82 genes that clustered with the GO term “nucleic acid transcription factor binding” (GO:0001071) and differed significantly between the samples from runners and nonrunners (*p* ≤ 0.05; Table S3). Within the Forkhead box (FOX) family of transcription factors, mRNAs for FoxG1, FoxA3, and FoxS1 were significantly upregulated (*p* ≤ 0.05). The FOX family proteins tune gene expression to regulate development and adult homeostasis, with roles in differentiation, metabolism, and proliferation [68]. FoxG1 is critical for differentiation of thymic epithelial cells, neural cells, and embryonic stem cells, and has roles within the nucleus, mitochondria, and cytoplasm [69–71]. For example, cellular metabolic state can be modified by changes in the compartmental localization of FoxG1[71]. FoxA3 regulates hematopoetic stem and progenitor cell survival and, in combination with tumor necrosis factor receptor 1, promotes liver regeneration [72, 73]. Using a FoxS1 knock-in reporter mouse, Heglind et al. [74] demonstrated FoxS1 expression in vascular smooth muscle cells and pericytes on the brain surface. FoxS1 is also expressed by pericytes and Sertoli cells in fetal testis and is required for the development of testicular vasculature [75]. FoxS1 also participates in a regulatory network that promotes fibroblast to myofibroblast differentiation [76].

Unexpectedly, in epicardial cells from runners we observed upregulation gene expression for a series of neuronal-associated transcription factors including: Neurodifferentiation 1 (NeuroD1; fold change = 1.27, *p*  < 0.001), Neurogenin 2 (Neurog2; fold change = 1.39, *p*  < 0.01), and Paired-box 3 (Pax3; fold change = 1.28, *p* ≤ 0.01; Table S3). NeuroD1 is a pioneering transcription factor that can uniquely access “closed” chromatin, thereby facilitating the binding activity of other transcriptional regulators [77].

#### 3.2.5. The Most Differentially Expressed Genes

Of the 4,112 differentially expressed transcripts, we identified by microarray expression, Snord116 increased the most in running-exercised mice (fold change = 7.08, *p*=0.044) and Igk-V28 decreased the most in running-exercised mice (fold change = −3.84, *p*=0.037). Transcripts with the largest fold change that were significantly different between running-exercised and control mice are shown in Table 1.

We were intrigued to find that running altered Snord116 expression, as it is primarily expressed by neurons in adults and had yet to be studied in epicardial cells. *SNORD116* (29 copies) resides within the highly conserved SNURF/SRPN lncRNA locus, a maternally imprinted (i.e., methylated) region located on chromosome 15 in humans (15q11.2-q13) and chromosome 7C in mice [78, 79]. Deletions, microdeletions, or DNA mutations in this chromosomal region cause PWS, a genetic disease characterized by the reduced levels of growth hormone, developmental delay, intellectual disability, sleep disorder, hyperphagia, and obesity [20, 78, 79]. Notably, microdeletions that remove *SNORD116* alone are sufficient to cause PWS in patients and mice with Snord116 deletion recapitulate salient features of PWS [20, 79].

The Snord116 gene contains noncoding “exons” and introns that undergo processing [80]. Snord116 exons are spliced into a lncRNA called Snord116HG that forms an RNA “cloud” near its site of transcription; this Snord116HG cloud was shown to control the diurnal expression of >2,400 metabolism-associated genes linked to energy expenditure [80]. In terms of cardiovascular health, some PWS patients have reduced capacity for exercise and exhibit microvascular dysfunction during stress tests [81]. Butler et al. [82] studied mitochondrial function in fibroblasts from PWS patients and control patients using Agilent Seahorse XF extracellular flux technology. They observed decreased mitochondrial function in fibroblasts from PWS patients compared to the control cells and reported significant differences in basal respiration, ATP-linked respiration, and maximal respiratory capacity [83].

#### 3.2.6. Snord116 Expression in Rat and Human Epicardial Cells

**Figure 4:**
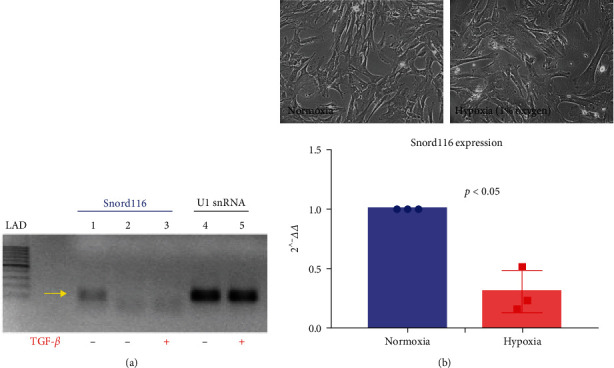
*Snord116* expresssion in rat epicardial cells and human EPDC. (a) TGF-*β*1 treatment of cultured adult rat epicardial cells decreased *Snord116* transcript levels. Lane 1, vehicle-treated cells, Snord116 probe/primers; lane 2, vehicle-treated cells, Snord116 probe/primers, no RT control; lane 3, TGF-*β*1-treated cells, Snord116 probe/primers; lane 4, vehicle-treated cells, U1 snRNA probe/primers; lane 5, TGF-*β*1-treated cells, U1 snRNA probe/primers. Note: U1 snRNA serves as a housekeeping gene for normalization of loading. (b) Top: phase contrast photomicrographs of primary human EPDC (passage 1) following EMT in medium containing 10% FCS. Cells were incubated under normoxic or hypoxic (1% oxygen) conditions for 48 hr. bottom: by real-time qRT-PCR assays, *Snord116* expression was significantly reduced under hypoxic conditions. *n* = 3 adult human donors.

We confirmed Snord116 expression in a specialized rat epicardial cell line that retains its epithelial character (Figure 4(a)). We found that 48-hr treatment with TGF-*β* (5 ng/mL), a factor which promotes epicardial cell EMT, reduced Snord116 expression (Figure 4(a)). Snord116 was also detected in primary human EPDC cultured in DMEM/F12 medium with 10% serum. Incubation of human EPDC under conditions of simulated ischemia (nutrient deprivation and 1% oxygen) for 48 hr significantly decreased Snord116 levels, as detected by qRT-PCR with validated Taqman probe and primers (*n* = 3 human donors, *p* < 0.05; Figure 4(b)). Together, these results confirmed Snord116 expression in epicardial cells from three different mammalian species.

### 3.3. Native Epicardial Cells Express FoxG1 In Vivo

**Figure 5:**
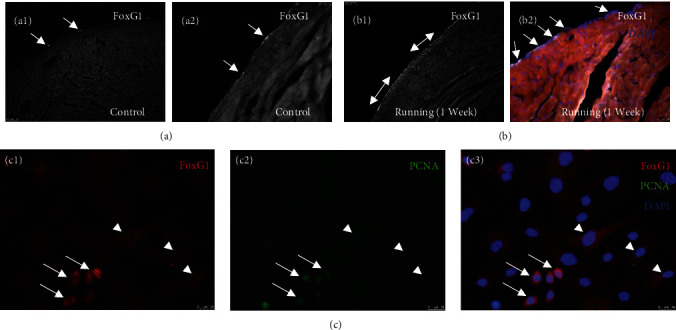
The transcription factor FoxG1 is expressed by epicardial cells *in vivo* and in culture. ((a): a1, a2) In nonrunning (control) mice, selected epicardial cells on the heart surface expressed FoxG1 (arrows). ((b): b1, b2) After 1 week of running exercise, FoxG1 is expressed by the majority of epicardial cells on the heart surface (arrows). Note: this observation agreed with microarray data showing increased levels of FoxG1 mRNA in CD104^+^ cells isolated from running-exercised mice. Bidirectional arrow indicate expression across the entire epicardium. (c1–c3) In culture, murine epicardial cells that highly expressed FoxG1 (see arrows) were positive also for nuclear-localized proliferating cell nuclear antigen (PCNA). Arrowheads label FoxG1-positive cells that are PCNA negative.

**Figure 6:**
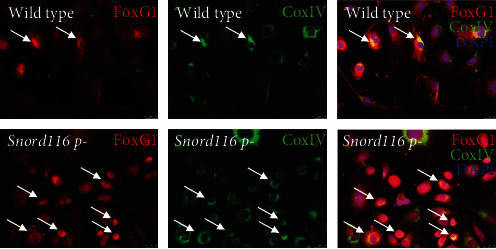
Paternal deletion of *Snord116* did not decrease FoxG1 expression or alter its localization in cultured murine epicardial cells. Epicardial cells from both wild type mice (top panels) and *Snord116 p-* mice (bottom panels) expressed FoxG1 in mitochondria (CoxIV, green) and nuclei (DAPI, blue). Yellow = colocalization (see arrows in panels on right). Prior to immunostaining, cells were cultured in LASR medium for 1 week.

Given that, FoxG1 exhibited the most significant change in expression among transcription factors (fold change = 1.53, *p* < 0.00001), we performed immunohistochemistry on tissue sections from hearts of control mice, running-exercised mice, and mice with MI. We observed sporadic epicardial FoxG1 staining on control hearts (Figures 5(a1) and 5(a2)). By contrast, hearts of running-exercised mice stained positively for FoxG1 in most cells along the entire epicardial surface (Figures 5(b1) and 5(b2)). FoxG1 was localized to epicardial cell nucleus and cytosol *in vivo* (Figure S2). After MI, antibodies to FoxG1 stained the surface epicardial cells, but not cells of the underlying subepicardium (Figure S3). To examine effects of Snord116 loss on FoxG1, we isolated epicardial cells from wild type and Snord116 p-mice and cultured them in LASR medium. Staining for cytochrome oxidase IV demonstrated that FoxG1 localized to mitochondria as well as nuclei (Figure 6). By immunohistochemical assays, Snord116 loss did not appear to reduce FoxG1 expression in the epicardial cells (Figure 6).

### 3.4. FoxG1 Controls Epicardial Cell Proliferation and Snord116 Expression

**Figure 7:**
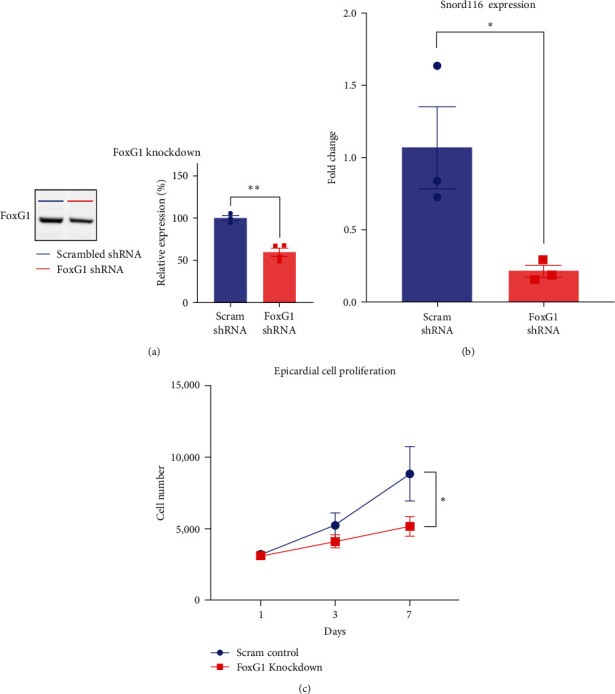
FoxG1 knockdown reduced *Snord116* expression and epicardial cell proliferation. (a) Left: lentiviral shRNA-mediated FoxG1 knockdown in rat epicardial cells, as shown by western blot. Right: quantification of FoxG1 protein demonstrated 40% knockdown compared with expression levels in control cells transduced with scrambled shRNA (*n* = 3–4 transduced cell populations, unpaired Student's *t*-test). (b) FoxG1 knockdown epicardial cell lines demonstrated a 79% decrease in *Snord116* expression compared with scrambled shRNA control cells (*n* = 3 transduced cell populations, unpaired Student's *t*-test). (c) FoxG1 knockdown significantly reduced epicardial cell proliferation. Two-way ANOVA with repeated measures and multiple comparisons. Mean ± SEM plotted in all graphs.  ^*∗*^*p*  < 0.05 and  ^*∗∗*^*p*  < 0.01.

FoxG1 shares transcriptional targets with Sox2 that include cell cycle regulators (e.g., Foxo3, Plk1, and Mycn) and epigenetic regulators (e.g., Dnmt1, Dnmt3b, and Tet3) [83]. Fluorescent immunocytochemical assays for FoxG1 and PCNA indicated that epicardial cells with FoxG1-positive nuclei were proliferating in culture (Figure 6(c)–6(e)). To determine effects of FoxG1 on epicardial cell proliferation, we used FoxG1-specific shRNA to knockdown FoxG1 protein levels in rat epicardial cell lines. Following lentiviral transduction and puromycin selection, western blot assays showed that the knockdown cell lines expressed significantly less FoxG1 compared with the level in cells transduced with scrambled control shRNA lentivector (Figure 7(a) and Figure S4). Also, by qRT-PCR assays, the knockdown cell lines had significantly reduced Snord116 expression (Figure 7(b)). Notably, FoxG1 knockdown significantly decreased epicardial cell proliferation compared to that in puromycin-selected control cell lines (Figure 7(c)). Further work with transcriptomics and genomics (e.g., single cell RNA-Seq, and ChIP-Seq) and functional studies with transgenic mice may further elucidate the roles of FoxG1 and Snord116 in the adult heart.

## 4. Conclusion

We used running-exercised mice as a model to study an adult epicardial gene expression during physiological (beneficial) cardiac remodeling. With microarray assays of cells isolated directly from runners and nonrunners, we identified *FoxG1* and *Snord116* as genes with elevated expression in epicardial cells of running mice. By immunochemistry, we observed epicardial FoxG1 expression *in vivo*, and in isolated proliferating mouse epicardial cells *in vitro*. FoxG1 knockdown resulted in decreased proliferation of the epicardial cells. Our results demonstrate that FoxG1 regulates epicardial growth and suggests that it may be an important factor for controlling cardiac remodeling.

## Figures and Tables

**Table 1:** Top upregulated and downregulated gene transcripts in CD104^+^ epicardial cells from running-exercised mice compared with genes expressed in CD104^+^ cells from nonrunners.

Up in running			Down in running		
Gene name	Fold change	Adjusted *p*-value	Gene name	Fold change	Adjusted *p*-value
Snord116	7.08	0.044	Igk-V28	−3.84	0.037
Mir101c	6.17	0.020	Zfp125	−3.13	0.008
Gm9602	4.91	0.034	Xlr3b	−3.12	0.440
Srsy	4.74	0.010	Gm13034	−3.04	0.031
Ssty2	4.22	0.041	Mirlet7f-1	−3.02	0.026
MGC107098	3.70	0.038	Snord61	−2.94	0.023
Gm2046	3.46	0.015	Omd	−2.64	0.008
Ssty1	3.05	0.001	Zfp932	−2.60	0.049
Sly	3.12	0.047	Pyhin1	−2.47	0.036

Cells were acutely isolated from hearts after 1 week of running exercise (ad libitum) or from hearts of aged-matched, nonrunning littermate controls. Our expression profiling analysis of epicardial cells identified 4,112 transcripts that were differentially expressed between cells of runners vs. nonrunners. Of these, 2,300 total transcripts were increased in runners and 1,812 total transcripts were decreased in runners (*p* ≤ 0.05).

## Data Availability

MicroArray data are available on ArrayExpress accession E-MTAB-10993.

## Supplementary Materials

Supplementary Materials Figure S1: immunocytochemistry of primary human epicardial cells. Compared with 3-week culture in DMEM/F12 medium containing 10% FCS, 3-week culture in LASR medium with Alk5 inhibitor markedly reduced the amount of EMT that occured over time. Whereas both cell populations expressed nuclear GATA4 (purple), the one cultured in LASR expressed higher levels of nuclear WT1 (red). By contrast, mesenchymal markers of EMT such as myosin light chain 2 (MLC2, green) were highly expressed in cultures maintained in DMEM/F12 medium with 10% FCS, but not so in cultures with LASR. Table S1: reproductive GO term changes (*p* ≤ 0.05). Table S2: extracellular matrix GO term changes (*p* ≤ 0.05). Table S3: nucleic acid binding transcription factor activity GO term changes (*p* ≤ 0.05). Figure S2: FoxG1 staining in (A) control and (B) running animals. FoxG1 was observed in the cytoplasm and nucleus (arrows) of epicardial cells after 8 weeks of running exercise (ad libitum) (B). Figure S3: expression of FoxG1 at 48 hr after myocardial infarction. FoxG1 is highly expressed in native epicardial cells after MI, particularly in epicardium residing adjacent to regions with infarction. These images are from different sections of the same mouse heart. Bidirectional arrows indicate expression across the entire epicardium. Figure S4: western blots demonstrate FoxG1 knockdown. Each blot shows three technical replicates from separately transfected cell populations (*n* = 4). FoxG1 KD = FoxG1 KnockDown.
