# Supplementary material for: The Neural Progenitor Cell-Associated Transcription Factor FoxG1 Regulates Cardiac Epicardial Cell Proliferation
Source: Stem Cells Int. 2024 Jan 11;2024:8601360. doi: 10.1155/2024/8601360 (PMC10796189; doi:10.1155/2024/8601360)
Supplement: Supplementary Materials — Figure S1: immunocytochemistry of primary human epicardial cells. Compared with 3-week culture in DMEM/F12 medium containing 10% FCS, 3-week culture in LASR medium with Alk5 inhibitor markedly reduced the amount of EMT that occured over time. Whereas both cell populations expressed nuclear GATA4 (purple), the one cultured in LASR expressed higher levels of nuclear WT1 (red). By contrast, mesenchymal markers of EMT such as myosin light chain 2 (MLC2, green) were highly expressed in cultures maintained in DMEM/F12 medium with 10% FCS, but not so in cultures with LASR. Table S1: reproductive GO term changes (p ≤ 0.05). Table S2: extracellular matrix GO term changes (p ≤ 0.05). Table S3: nucleic acid binding transcription factor activity GO term changes (p ≤ 0.05). Figure S2: FoxG1 staining in (A) control and (B) running animals. FoxG1 was observed in the cytoplasm and nucleus (arrows) of epicardial cells after 8 weeks of running exercise (ad libitum) (B). Figure S3: expression of FoxG1 at 48 hr after myocardial infarction. FoxG1 is highly expressed in native epicardial cells after MI, particularly in epicardium residing adjacent to regions with infarction. These images are from different sections of the same mouse heart. Bidirectional arrows indicate expression across the entire epicardium. Figure S4: western blots demonstrate FoxG1 knockdown. Each blot shows three technical replicates from separately transfected cell populations (n = 4). FoxG1 KD = FoxG1 KnockDown. [file 8601360.f1.docx]

Supplementary Material

**The neural progenitor cell-associated transcription factor FoxG1 regulates cardiac epicardial cell proliferation.**

**Lucy Pilcher^1,2†^, Lara Solomon^1,2†^, Julie A. Dragon^3^, Dhananjay Gupta^4^, and Jeffrey L. Spees^1,2*^**

*** Correspondence:** [jspees@uvm.edu](mailto:Jeffrey.Spees@uvm.edu)

# Supplementary Methods and Materials

Myocardial infarction (MI) in mice

MI was induced in immunocompetent mice (C57 Bl/6J background, males, 8-10 weeks of age) under general anesthesia (isoflurane, 1-4% to effect). Through a left thoracotomy, the mid left anterior descending (LAD) coronary artery was permanently ligated (as in Rao et al., 2015). Full sham surgery controls were performed, in which the suture was passed underneath the LAD, but not ligated. All animal work was conducted in accordance with protocols approved by the Institutional Animal Care and Use Committee (IACUC) of the University of Vermont.

Isolation of mouse epicardial cells

Mice were euthanized by CO_2_ and cervical dislocation. Mice were then moved to a sterile hood and wiped with 70% ethanol. The skin and muscle were cut along the bottom of the rib cage and the diaphragm was cut along the bottom of the sternum and ribs to expose the heart. The ribs were then cut along each side on the animal. Using a 27-gauge needle, we perfused hearts through the left ventricle with 20 mL PBS (Ca^2+^ and Mg^2+^ free). The heart was excised from the aorta and rinsed in 15 mL PBS. The heart was then moved to 15 mL of HBSS for up to 30 minutes. The hearts were digested for 2 hours at 37 degrees Celsius with gentle shaking. Digest solution was 5 mg/ml Collagenase/Dispase (Roche Diagnostics) and 10 μM Cyclosporin A (Caymen Chemical) in HBSS. The digest solution was gently pipetted over the heart twice and collected. The heart was rinsed twice with PBS. Total digest and rinse solution was collected into a 50 mL centrifuge tube and 5 mL of MACS Buffer was used to triturate the suspension 5 times. MACS Buffer: 2 mM EDTA (Sigma Aldrich) and 0.5% Biotin-free BSA (Sigma) in DMEM/F12. The suspension was centrifuged for 8 minutes at 800 g, and re-suspended in 500 μL MACS buffer by gentle pipetting. Then 5 μL anti-mouse CD104 (AbDSerotec) was added and the cells were incubated on ice for 30 minutes, with gentle agitation every 5 minutes. Cells were washed with 25 mL of MACS buffer and 5 inversions, spun at 800 x g for 8 minutes, then re-suspended in 200 μL MACS buffer and 50 μL anti-rat micro-beads (Miltenyi Biotech). The cells were again incubated on ice with 30 minutes on ice with gentle agitation every 5 minutes. Cells were washed with 25 mL MACS buffer and 5 inversions, spun at 800 g for 8 minutes, then re-suspended in 200 μL and counted. Then, the cells were re-suspended in 5 mL MACS buffer. The LS columns were pre-rinsed with 5 mL MACS Buffer, and cell suspension was added to the column. The column was rinsed with at least 15 mL MACS buffer before the column was removed from the magnet. Cells were collected in 5 mL MACS Buffer. Cells were spun at 800 x g for 8 minutes and re-suspended in 200 μL MACS buffer for counting. The cells were finally spun for 8 minutes at 800 x g.

RT-qPCR of Snord116 in human EPDCs

Human EPDCs from right atrial biopsies were cultured in DMEM/F12 with 10% serum. Plates were grown to 100% confluence and incubated in normoxic conditions or hypoxic conditions for 24-48 hours prior to harvest. RNA was isolated using Zymo QuickRNA miniPrep Plus kit according to manufacturer instructions, treated with TurboDnase (Invitrogen), and quantified on a Nanodrop 1000. cDNA was produced using 1 μg of RNA in a 20 μL reaction of Superscript III reverse transcriptase and random hexamers (Invitrogen). 2 μL of the cDNA reaction was used per well for qPCR using Taqman fast advanced master mix and TaqMan assays (Snord116: Hs03309547_s1 and ScarnA5: Hs03298717_s1).

**Echocardiography**
Two dimensional, Doppler, and M-Mode echocardiography was performed with a Vevo770 High-Resolution Imaging System (VisualSonics, Toronto, ON, Canada) by an operator blinded to the animal groups. Echocardiography was performed with mice first anesthetized with isoflurane. Animals were then weighed and placed supine onto a heated imaging platform. The distal extremities were taped gently to electro pads that provide continuous electrocardiographic and hear rate (pulse detection) measurements. Immediately before imaging, Nair (hair removal cream) was applied with a Q-tip. Warmed gel used for ultrasonic studies was placed on the chest, and the 60 MHz probe is lowered onto the gel. All left ventricular dimensions in systole and diastole were measured from M-mode images obtained at the mid-papillary muscle level.

# Supplementary Figures


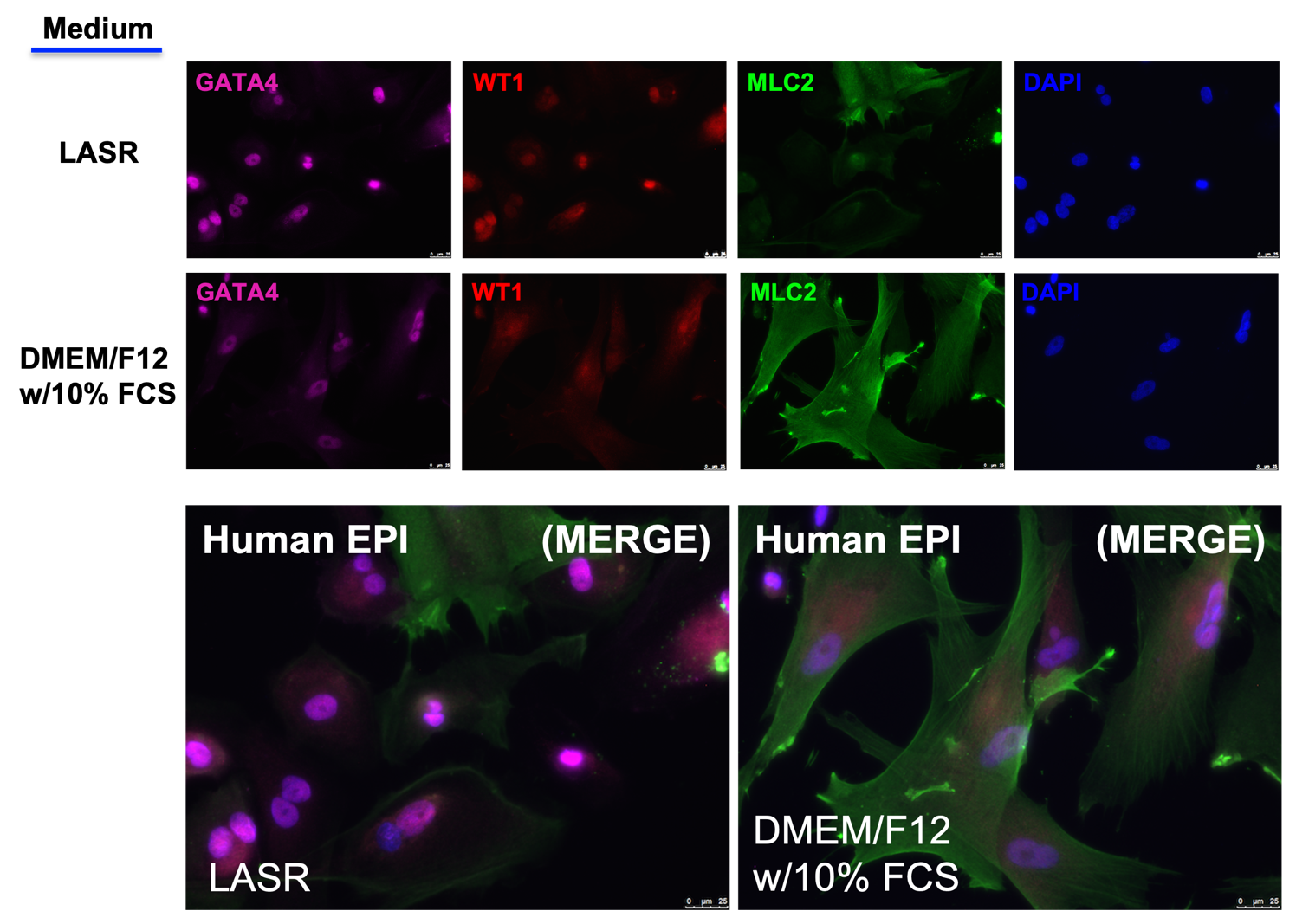


**Supplemental Figure 1.** Immunocytochemistry of primary human epicardial cells. Compared with 3 week culture in DMEM/F12 medium containing 10% FCS, 3 week culture in LASR medium with Alk5 inhibitor markedly reduced the amount of EMT that occured over time. Whereas both cell populations expressed nuclear GATA4 (purple), the one cultured in LASR expressed higher levels of nuclear WT1 (red). By contrast, mesenchymal markers of EMT such as myosin light chain 2 (MLC2, green) were highly expressed in cultures maintained in DMEM/F12 medium with 10% FCS, but not so in cultures with LASR.

**
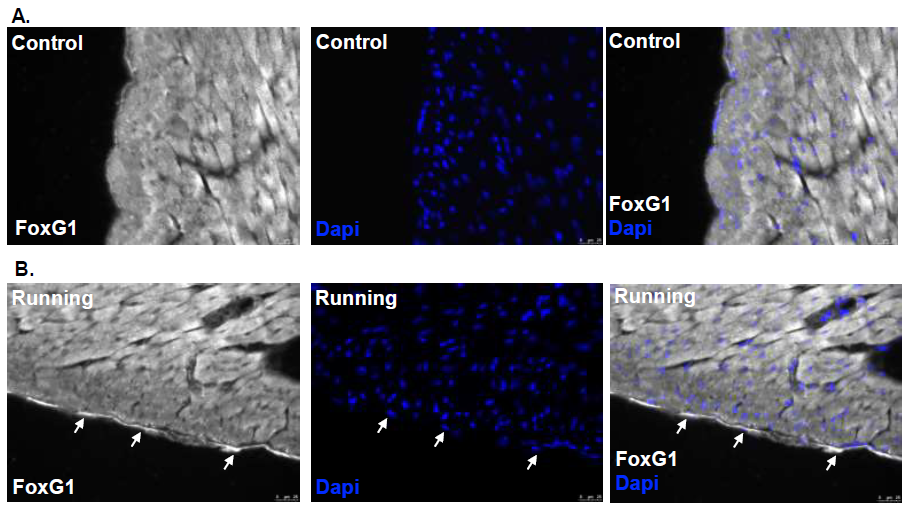
Supplemental Figure 2.** FoxG1 staining in **(A)** control and **(B)** running animals. FoxG1 was observed in the cytoplasm and nucleus (arrows) of epicardial cells after 8 weeks of running exercise (ad libitum) (B).


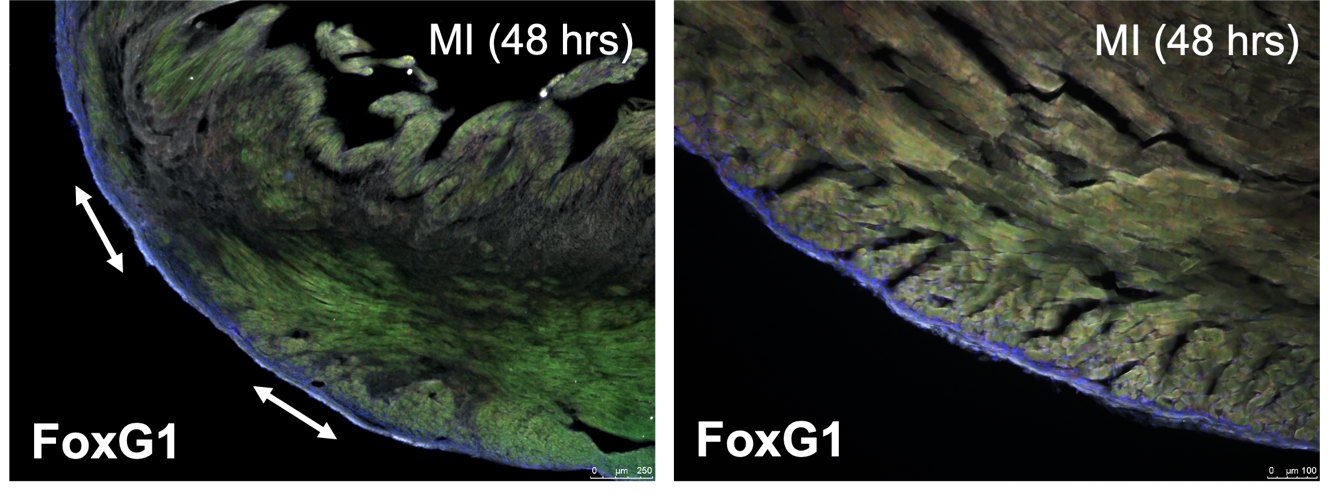
**Supplemental Figure 3.** Expression of FoxG1 at 48 hours after myocardial infarction. FoxG1 is highly expressed in native epicardial cells after MI, particularly in epicardium residing adjacent to regions with infarction. These images are from different sections of the same mouse heart. Bidirectional arrows indicate expression across the entire epicardium.

# Supplementary Tables

Supplemental Table 1. Reproductive GO term changes (p ≤ 0.05).

Please note that "R" refers to running samples, whereas "C" refers to control samples.

| Gene Symbol | RefSeq | p-value | Fold-change | Description |
| --- | --- | --- | --- | --- |
| Bcl2 | NM_009741 | 0.000337 | -1.26863 | R down vs C |
| Spin4 | NM_178753 | 0.00045 | -1.35393 | R down vs C |
| Prdx4 | NM_016764 | 0.000473 | -1.61472 | R down vs C |
| Ddx4 | NM_001145885 | 0.000878 | 1.23375 | R up vs C |
| Immp2l | ENSMUST00000134965 | 0.001299 | -1.40526 | R down vs C |
| Lrp6 | NM_008514 | 0.001843 | 1.12648 | R up vs C |
| Atp8b3 | NM_026094 | 0.00206 | 1.20365 | R up vs C |
| Syce1 | NM_001143765 | 0.002348 | 1.1989 | R up vs C |
| Cul7 | NM_025611 | 0.002531 | 1.19327 | R up vs C |
| Nr2f2 | NM_183261 | 0.004946 | 1.1682 | R up vs C |
| Meig1 | ENSMUST00000115083 | 0.005948 | -1.6268 | R down vs C |
| Jag2 | NM_010588 | 0.006431 | 1.11351 | R up vs C |
| Th | ENSMUST00000000219 | 0.007041 | 1.21415 | R up vs C |
| Map2k1 | NM_008927 | 0.007998 | -1.16327 | R down vs C |
| Birc6 | NM_007566 | 0.00851 | -1.11453 | R down vs C |
| Plekha1 | NM_133942 | 0.009155 | -1.24818 | R down vs C |
| Corin | NM_016869 | 0.010197 | 1.20051 | R up vs C |
| Ift81 | NM_009879 | 0.010946 | -1.61298 | R down vs C |
| Angpt1 | ENSMUST00000022921 | 0.0113 | -1.84062 | R down vs C |
| Dicer1 | NM_148948 | 0.011597 | -1.20149 | R down vs C |
| Cdh1 | NM_009864 | 0.011853 | -1.22617 | R down vs C |
| Exo1 | NM_012012 | 0.011946 | 1.54586 | R up vs C |
| Atm | NM_007499 | 0.01197 | -1.41555 | R down vs C |
| Zfp37 | NM_009554 | 0.013281 | 1.09552 | R up vs C |
| Rad23b | NM_009011 | 0.013461 | -1.13956 | R down vs C |
| Fancl | ENSMUST00000004120 | 0.01347 | -1.47642 | R down vs C |
| Smc3 | NM_007790 | 0.013632 | -1.17291 | R down vs C |
| Rps6 | NM_009096 | 0.014052 | -1.03026 | R down vs C |
| Rad21l | NM_001114677 | 0.014082 | 1.21252 | R up vs C |
| Kitl | ENSMUST00000105283 | 0.014372 | -1.24251 | R down vs C |
| Tle3 | NM_001083927 | 0.014802 | 1.12531 | R up vs C |
| Tiparp | NM_178892 | 0.015107 | -1.56611 | R down vs C |
| Stk3 | NM_019635 | 0.015633 | -1.42692 | R down vs C |
| Dld | NM_007861 | 0.015677 | -1.351 | R down vs C |
| Golga3 | ENSMUST00000112512 | 0.015776 | -1.28204 | R down vs C |
| Bptf | NM_176850 | 0.016261 | -1.09183 | R down vs C |
| Lgr5 | NM_010195 | 0.016527 | -1.2999 | R down vs C |
| Src | ENSMUST00000109533 | 0.017835 | -1.18975 | R down vs C |

(Con’t) Supplemental Table 1. Reproductive GO term changes (p ≤ 0.05)

| Gene Symbol | RefSeq | p-value | Fold-Change | Description |
| --- | --- | --- | --- | --- |
| Aurka | NM_011497 | 0.017905 | 1.48953 | R up vs C |
| Rnase9 | NM_183032 | 0.0182823 | 1.29313 | R up vs C |
| Mir34b | NR_029655 | 0.0183713 | 1.76618 | R up vs C |
| Chd7 | ENSMUST00000051558 | 0.0184249 | -1.13204 | R down vs C |
| Magoh | ENSMUST00000030348 | 0.0189665 | -1.38423 | R down vs C |
| Ttc26 | ENSMUST00000162554 | 0.0195192 | -1.24241 | R down vs C |
| Ift88 | NM_009376 | 0.0198661 | -1.21459 | R down vs C |
| Stag3 | NM_016964 | 0.0202322 | 1.59994 | R up vs C |
| Foxa3 | NM_008260 | 0.0214774 | 1.37347 | R up vs C |
| Plk1 | NM_011121 | 0.0215838 | 1.34933 | R up vs C |
| Tubd1 | NM_001199045 | 0.0220877 | -1.47585 | R down vs C |
| Gm16405 | NM_001166646 | 0.022274 | 1.62965 | R up vs C |
| Sos1 | NM_009231 | 0.0236532 | -1.15704 | R down vs C |
| Gm16405 | NM_001166646 | 0.024036 | 1.63986 | R up vs C |
| Tial1 | ENSMUST00000106226 | 0.0240807 | -1.2188 | R down vs C |
| Spata18 | ENSMUST00000071077 | 0.0241633 | 1.29712 | R up vs C |
| Mlh3 | NM_175337 | 0.0241823 | -1.52851 | R down vs C |
| Mnd1 | NM_029797 | 0.0255297 | -1.71702 | R down vs C |
| Larp7 | NM_138593 | 0.0256227 | -1.24027 | R down vs C |
| Top2a | NM_011623 | 0.0256785 | 1.26988 | R up vs C |
| Dnajb6 | ENSMUST00000008733 | 0.0264316 | -1.1576 | R down vs C |
| Ggn | NM_182694 | 0.0273 | 1.19814 | R up vs C |
| Mir449c | NR_030452 | 0.0281433 | 1.38545 | R up vs C |
| Mlh1 | NM_026810 | 0.0284702 | -1.22918 | R down vs C |
| Strbp | NM_009261 | 0.0289145 | -1.20595 | R down vs C |
| Fzd5 | NM_022721 | 0.0289334 | 1.04561 | R up vs C |
| Spaca3 | ENSMUST00000103223 | 0.0298774 | 1.24536 | R up vs C |
| Edn2 | ENSMUST00000030384 | 0.0302254 | 1.12367 | R up vs C |
| Tdrd7 | ENSMUST00000102929 | 0.0306424 | -1.21717 | R down vs C |
| Adam26a | NM_010085 | 0.0309436 | 1.14646 | R up vs C |
| Ror2 | NM_013846 | 0.0312218 | 1.29694 | R up vs C |
| Racgap1 | NM_012025 | 0.0317615 | 1.349 | R up vs C |
| Tbata | NM_001017433 | 0.0323685 | 1.31109 | R up vs C |
| Spin1 | NM_011462 | 0.0330707 | -1.27487 | R down vs C |
| Trp63 | NM_001127259 | 0.0331425 | 1.21736 | R up vs C |
| Gm16405 | NM_001166646 | 0.0345515 | 1.56139 | R up vs C |
| Klf17 | NM_029416 | 0.0345984 | 1.17917 | R up vs C |
| Rbm7 | NR_037589 | 0.0347037 | -1.355 | R down vs C |
| Arid4a | NM_001081195 | 0.0347856 | -1.25435 | R down vs C |
| Prss29 | NM_053260 | 0.0352826 | 1.32047 | R up vs C |
| Bmpr1b | NM_007560 | 0.0355947 | -1.41594 | R down vs C |

(Con’t) Supplemental Table 1. Reproductive GO term changes (p ≤ 0.05)

| Gene Symbol | RefSeq | p-value | Fold-Change | Description |
| --- | --- | --- | --- | --- |
| Senp2 | NR_027488 | 0.0356673 | -1.36295 | R down vs C |
| Tcp1 | ENSMUST00000129632 | 0.0363534 | -1.36122 | R down vs C |
| Xlr5b | ENSMUST00000114518 | 0.0365923 | 1.25332 | R up vs C |
| Ccdc155 | ENSMUST00000121017 | 0.036816 | 1.2357 | R up vs C |
| Xlr5a | NM_001045539 | 0.0382874 | 1.34647 | R up vs C |
| Insl6 | NM_013754 | 0.0383083 | -1.36024 | R down vs C |
| Mastl | ENSMUST00000028119 | 0.0388907 | -1.24928 | R down vs C |
| Zpbp | ENSMUST00000020413 | 0.0395269 | -1.22044 | R down vs C |
| Nme5 | NM_080637 | 0.039912 | -1.98329 | R down vs C |
| Nrip1 | ENSMUST00000121927 | 0.0405245 | -1.14789 | R down vs C |
| 3830403N18Rik | NM_027510 | 0.0413558 | -1.5917 | R down vs C |
| Ggt1 | ENSMUST00000006508 | 0.0413616 | 1.24216 | R up vs C |
| Ube2a | NM_019668 | 0.0428619 | -1.43152 | R down vs C |
| Zfx | NM_001044386 | 0.0439853 | -1.29493 | R down vs C |
| Xlr3b | NM_001081643 | 0.0441813 | -3.1188 | R down vs C |
| Dmrtc2 | NM_027732 | 0.0443115 | 1.3555 | R up vs C |
| Gm16405 | NM_001166646 | 0.0464341 | 1.52703 | R up vs C |
| Myocd | NM_145136 | 0.0464727 | 1.24754 | R up vs C |
| Klhdc3 | NM_027910 | 0.0466139 | -1.22938 | R down vs C |
| Sly | NM_201530 | 0.0474687 | 3.1055 | R up vs C |
| Sly | NM_201530 | 0.0474687 | 3.1055 | R up vs C |
| Plcb1 | NM_001145830 | 0.0475432 | -1.22738 | R down vs C |
| Syna | NM_001013751 | 0.047634 | 1.21241 | R up vs C |
| Ubr2 | NM_146078 | 0.048423 | -1.1173 | R down vs C |
| Tmf1 | NM_001081111 | 0.0487619 | -1.29388 | R down vs C |
| Pank2 | NM_153501 | 0.0489449 | -1.32359 | R down vs C |
| Gm16405 | NM_001166646 | 0.0493263 | 1.56571 | R up vs C |
| Gm16405 | NM_001166646 | 0.0493263 | 1.56571 | R up vs C |
| Ada | ENSMUST00000017841 | 0.0496975 | -1.10595 | R down vs C |
| Rpa1 | NM_001164223 | 0.0498222 | -1.18088 | R down vs C |
| Cr1l | NM_013499 | 0.0498539 | -1.21465 | R down vs C |
| Esx1 | NM_007957 | 0.0510489 | 1.06804 | R up vs C |
| Rps6kb1 | ENSMUST00000154617 | 0.051276 | -1.20501 | R down vs C |
| Cct4 | ENSMUST00000173867 | 0.0516874 | -1.29942 | R down vs C |
| H3f3a | NM_008210 | 0.0520766 | -1.13347 | R down vs C |
| Rsl1 | NM_001013769 | 0.0525729 | -1.99801 | R down vs C |
| Camk2b | NM_007595 | 0.0531682 | 1.46717 | R up vs C |
| Ubb | ENSMUST00000019649 | 0.0537239 | -1.07926 | R down vs C |
| Dpcd | NM_172639 | 0.0541406 | -1.1721 | R down vs C |
| Spire2 | NM_172287 | 0.0542897 | 1.30056 | R up vs C |

Supplemental Table 2. Extracellular matrix GO term changes (p ≤ 0.05).

Please note that "R" refers to running samples, whereas "C" refers to control samples.

| **Gene Symbol** | **RefSeq** | **p-value** | **Fold-Change** | **Description** |
| --- | --- | --- | --- | --- |
| Nyx | ENSMUST00000050434 | 0.000353713 | 1.07561 | R up vs C |
| Adamts19 | NM_175506 | 0.00485991 | -1.2845 | R down vs C |
| Fgfbp3 | NM_028263 | 0.00621279 | 1.34054 | R up vs C |
| Adamtsl4 | ENSMUST00000117782 | 0.00691365 | 1.11175 | R up vs C |
| Omd | NM_012050 | 0.0084728 | -2.64049 | R down vs C |
| Ltbp4 | NM_175641 | 0.008994 | 1.14815 | R up vs C |
| Col9a1 | NM_007740 | 0.0129199 | 1.16006 | R up vs C |
| F2 | NM_010168 | 0.0177218 | 1.18622 | R up vs C |
| Mmp13 | NM_008607 | 0.0181789 | -1.39431 | R down vs C |
| Vit | NM_028813 | 0.0181927 | -1.73122 | R down vs C |
| Col7a1 | NM_007738 | 0.0188026 | 1.31585 | R up vs C |
| Smoc2 | NM_022315 | 0.0193972 | -1.43192 | R down vs C |
| Lad1 | NM_133664 | 0.0221591 | 1.35419 | R up vs C |
| Mmp3 | NM_010809 | 0.0232333 | -1.6688 | R down vs C |
| Crtac1 | NM_145123 | 0.0240654 | 1.0597 | R up vs C |
| Col1a1 | ENSMUST00000001547 | 0.0243705 | 1.26197 | R up vs C |
| Pkm | NM_011099 | 0.0262531 | 1.14644 | R up vs C |
| Mmp19 | NM_021412 | 0.026869 | -1.26095 | R down vs C |
| Hpse | NM_152803 | 0.0274543 | -1.20961 | R down vs C |
| Mmp16 | ENSMUST00000029881 | 0.030132 | -1.10665 | R down vs C |
| Dcn | NM_007833 | 0.0306457 | -1.08987 | R down vs C |
| Impg2 | NM_174876 | 0.0311182 | 1.18478 | R up vs C |
| Col19a1 | NM_007733 | 0.0315979 | 1.172 | R up vs C |
| Tnn | NM_177839 | 0.0344764 | 1.63442 | R up vs C |
| Gpc5 | NM_175500 | 0.0349341 | 1.49155 | R up vs C |
| Ptn | NM_008973 | 0.0350216 | -1.59893 | R down vs C |
| Matn2 | NM_016762 | 0.0367851 | -1.21501 | R down vs C |
| Ncan | NM_007789 | 0.0371924 | 1.51546 | R up vs C |
| Col6a1 | ENSMUST00000105412 | 0.0399645 | -1.2992 | R down vs C |
| Nav2 | NM_175272 | 0.0423978 | 1.04991 | R up vs C |
| Wisp1 | NM_018865 | 0.0435078 | 1.24811 | R up vs C |
| Egfl6 | NM_019397 | 0.0439293 | -1.22253 | R down vs C |
| Ogn | NM_008760 | 0.0457683 | -1.40855 | R down vs C |
| Tff3 | ENSMUST00000024827 | 0.0479026 | 1.16069 | R up vs C |
| Adamtsl1 | NM_029967 | 0.0479986 | -1.09988 | R down vs C |
| B4galt7 | NM_146045 | 0.0527687 | 1.46157 | R up vs C |

**Supplemental Table 3**. Nucleic acid binding transcription factor activity GO term changes (p ≤ 0.05). Please note that "R" refers to running samples, whereas "C" refers to control samples.

| **Gene Symbol** | **RefSeq** | **p-value** | **Fold-Change** | **Description** |
| --- | --- | --- | --- | --- |
| Foxg1 | NM_001160112 | 8.45665E-05 | 1.52685 | R up vs C |
| Cebpz | NM_001024806 | 0.00028732 | -1.19364 | R down vs C |
| Neurod1 | ENSMUST00000041099 | 0.000969303 | 1.2716 | R up vs C |
| Cebpz | NM_001024806 | 0.00127789 | -1.24735 | R down vs C |
| Gm13212 | ENSMUST00000105739 | 0.00212063 | 1.36206 | R up vs C |
| Gm13152 | ENSMUST00000063704 | 0.00253476 | -1.64509 | R down vs C |
| Gm10324 | NM_001177832 | 0.00287083 | 2.01704 | R up vs C |
| Zfp260 | ENSMUST00000050735 | 0.00318541 | -1.29374 | R down vs C |
| Zfp280c | NM_153532 | 0.00327805 | -1.41053 | R down vs C |
| Zfp873 | NM_001024626 | 0.0034278 | -1.71725 | R down vs C |
| Nr2f2 | NM_183261 | 0.00494576 | 1.1682 | R up vs C |
| Pydc3 | NM_001162938 | 0.0051313 | 1.30067 | R up vs C |
| Nfix | NM_001081982 | 0.00516407 | 1.28071 | R up vs C |
| Taf7 | NM_175770 | 0.00531101 | -1.54954 | R down vs C |
| Zfp292 | NM_013889 | 0.00552803 | -1.20545 | R down vs C |
| Trps1 | NM_032000 | 0.0059242 | -1.20386 | R down vs C |
| Hivep2 | NM_010437 | 0.00644032 | -1.24902 | R down vs C |
| Tfap4 | NM_031182 | 0.00672151 | -1.18639 | R down vs C |
| Nrf1 | NM_001164226 | 0.00677603 | -1.27223 | R down vs C |
| Pou2f1 | NM_011137 | 0.00688473 | -1.28722 | R down vs C |
| Spen | NM_019763 | 0.00751398 | 1.08036 | R up vs C |
| Nfe2l3 | NM_010903 | 0.00763292 | -1.18432 | R down vs C |
| Mybl2 | ENSMUST00000137620 | 0.00786876 | 1.31783 | R up vs C |
| Zkscan16 | NM_001099323 | 0.00922455 | 1.18695 | R up vs C |
| Tbp | ENSMUST00000162505 | 0.0107141 | -1.30174 | R down vs C |
| Phox2b | ENSMUST00000012664 | 0.0114535 | 1.47172 | R up vs C |
| Nr1d2 | NM_011584 | 0.0133043 | -1.08283 | R down vs C |
| Pax3 | NM_008781 | 0.0136547 | 1.28485 | R up vs C |
| Hnf4g | NM_013920 | 0.015026 | 1.47782 | R up vs C |
| Nfyb | NM_010914 | 0.015417 | -1.61425 | R down vs C |
| Max | NM_008558 | 0.0157094 | 1.0251 | R up vs C |
| Six3 | ENSMUST00000162989 | 0.015983 | 1.25032 | R up vs C |
| Zfp454 | ENSMUST00000163301 | 0.0197601 | 1.41293 | R up vs C |
| Strn3 | NM_052973 | 0.0199519 | -1.28384 | R down vs C |
| Tbr1 | NM_009322 | 0.020372 | 1.04961 | R up vs C |
| Zfp583 | NM_001033249 | 0.0210021 | 1.09634 | R up vs C |
| Zscan4f | NM_001110316 | 0.021287 | 1.3592 | R up vs C |
| Foxa3 | NM_008260 | 0.0214774 | 1.37347 | R up vs C |

(Con’t) Supplemental Table 3. Nucleic acid binding transcription factor activity GO term changes (p ≤ 0.05)

| **Gene Symbol** | **RefSeq** | **p-value** | **Fold-Change** | **Description** |
| --- | --- | --- | --- | --- |
| Rfx1 | NM_009055 | 0.0217291 | 1.33907 | R up vs C |
| Zfp738 | NM_001001187 | 0.0222254 | -1.87227 | R down vs C |
| Zfp26 | NM_011753 | 0.0228167 | 1.10204 | R up vs C |
| Gm3604 | NM_001162910 | 0.0237986 | -2.26011 | R down vs C |
| Tfdp2 | NM_178667 | 0.0244442 | -1.13573 | R down vs C |
| Rreb1 | NR_033218 | 0.0256583 | 1.13678 | R up vs C |
| Rfx8 | NM_001145660 | 0.0269763 | 1.09434 | R up vs C |
| Myod1 | NM_010866 | 0.0278803 | 1.32405 | R up vs C |
| Rcor1 | ENSMUST00000084968 | 0.0281383 | -1.44755 | R down vs C |
| Creb1 | NM_133828 | 0.028558 | -1.18364 | R down vs C |
| Zfp708 | NM_001012448 | 0.0293226 | -1.9409 | R down vs C |
| Mndal | NM_001170853 | 0.029535 | -1.70056 | R down vs C |
| Barhl2 | ENSMUST00000086795 | 0.0299535 | 1.12684 | R up vs C |
| Sox4 | ENSMUST00000067230 | 0.0303399 | -1.21826 | R down vs C |
| Zfp758 | NM_145484 | 0.0329337 | -1.77883 | R down vs C |
| Trp63 | NM_001127259 | 0.0331425 | 1.21736 | R up vs C |
| Smad2 | NM_001252481 | 0.033868 | -1.1869 | R down vs C |
| Onecut2 | NM_194268 | 0.0340372 | 1.50857 | R up vs C |
| Klf17 | NM_029416 | 0.0345984 | 1.17917 | R up vs C |
| Pyhin1 | NM_175026 | 0.036212 | -2.46849 | R down vs C |
| Foxs1 | NM_010226 | 0.0366845 | -1.37471 | R down vs C |
| Hoxa3 | ENSMUST00000114434 | 0.0373524 | 1.44064 | R up vs C |
| Mef2b | NM_008578 | 0.0373947 | 1.4804 | R up vs C |
| C030039L03Rik | NM_198417 | 0.039011 | 1.41853 | R up vs C |
| Foxd4 | NM_008022 | 0.0395271 | 1.17199 | R up vs C |
| E2f2 | NM_177733 | 0.0400394 | 1.61577 | R up vs C |
| Zfp954 | NM_172738 | 0.0403186 | -1.88372 | R down vs C |
| Myt1l | NM_001093775 | 0.0403235 | 1.39268 | R up vs C |
| Gm14431 | NM_001177406 | 0.0406031 | -1.25388 | R down vs C |
| Zfp72 | NM_001081680 | 0.0409253 | -1.25103 | R down vs C |
| Sp4 | NM_009239 | 0.0409446 | -1.426 | R down vs C |
| Msgn1 | NM_019544 | 0.0415572 | 1.65395 | R up vs C |
| Zfp280d | BC027163 | 0.0422592 | -1.16838 | R down vs C |
| Gpbp1 | ENSMUST00000047627 | 0.0428043 | -1.41416 | R down vs C |
| Sall4 | NM_175303 | 0.042807 | 1.47252 | R up vs C |
| E2f5 | NM_007892 | 0.0433716 | -1.16136 | R down vs C |
| Zfx | NM_001044386 | 0.0439853 | -1.29493 | R down vs C |
| Dmrtc2 | NM_027732 | 0.0443115 | 1.3555 | R up vs C |

(Con’t) Supplemental Table 3. Nucleic acid binding transcription factor activity GO term changes (p ≤ 0.05)

| **Gene Symbol** | **RefSeq** | **p-value** | **Fold-Change** | **Description** |
| --- | --- | --- | --- | --- |
| 2410141K09Rik | NM_183119 | 0.0443612 | 1.65326 | R up vs C |
| Bhlha15 | NM_010800 | 0.0451731 | 1.47976 | R up vs C |
| Klf7 | ENSMUST00000114086 | 0.045181 | -1.58596 | R down vs C |
| Myocd | NM_145136 | 0.0464727 | 1.24754 | R up vs C |
| Zfp420 | NM_172740 | 0.0484832 | -1.46786 | R down vs C |
| Zfp174 | NM_001081217 | 0.0487177 | 1.39146 | R up vs C |
| Tfap2a | NM_011547 | 0.0498445 | 1.22812 | R up vs C |
| Rarb | ENSMUST00000063750 | 0.0508782 | -1.33049 | R down vs C |
| Zfp68 | NM_013844 | 0.0513493 | -2.41467 | R down vs C |
| Mnda | NM_001033450 | 0.052145 | -1.29558 | R down vs C |
| Crebzf | NM_145151 | 0.0521686 | -1.26625 | R down vs C |
| Mkl2 | NM_001122667 | 0.0522084 | 1.09102 | R up vs C |
| Esrrg | ENSMUST00000110938 | 0.0522817 | 1.10716 | R up vs C |
| Hltf | NM_009210 | 0.0523253 | -1.68545 | R down vs C |
| Tfap2d | NM_153154 | 0.052531 | 1.07777 | R up vs C |
| Sarnp | NM_025364 | 0.0542368 | -1.72586 | R down vs C |
